# The association between early onset of alcohol, smokeless tobacco and marijuana use with adult binge drinking in United States

**DOI:** 10.1038/s41598-023-27571-x

**Published:** 2023-01-05

**Authors:** Zheng Dai, Kesheng Wang

**Affiliations:** 1 Health Affairs Institute, West Virginia University, Morgantown, USA; 2 Department of Family and Community Health, School of Nursing, West Virginia University, Morgantown, USA

**Keywords:** Diseases, Risk factors, Epidemiology

## Abstract

Binge drinking is a deadly pattern of excessive alcohol use that is associated with multiple diseases in the United States. To date, little is known about the associations between the early onset of substance use and other factors with the severity of adult binge drinking. The 2018 National Survey on Drug Use and Health data was used to identify binge drinking (binary and in number of days in the past month). Age at onset was categorized into four groups as 1–12, 13–14, 15–17, or beyond 18. Weighted multivariate logistic regression and Poisson regression analyses were performed to examine the associations between early onset of alcohol, smokeless tobacco, and marijuana use with binge drinking. The severity of binge drinking was statistically significantly associated with substance use (4.15 days in a month), early onset of alcohol, smokeless tobacco, and marijuana use (2.15–4.93 days, all p-values < 0.0001), after accounting for the covariates. Past year substance use disorder is strongly associated with binge drinking. The severity of adult binge drinking is significantly associated with early onset of substance use including alcohol, smokeless tobacco, and marijuana. Continued efforts are warranted to improve substance use prevention and treatment tailored for adolescents and youths to prevent development of adult binge drinking.

## Introduction

Previous studies have shown that binge drinking was strongly associated with a wide range of serious health outcomes, including all-cause mortality^1,2^, most prevalent types of cancer^3,4^, serious psychological distress (SPD)^5,6^, and multiple chronic conditions^7–11^. According to the 2019 National Survey on Drug Use and Health (NSDUH) data, almost 30% of adults ages 18 and older reported binge drinking in the past month^12^. The majority of previous studies, including some large-scale studies^13^, have established that early onset of drinking is a known risk factor for adulthood heavy drinking^14–16^. However, some inconsistent study findings suggested little to no evidence for this association, which might be contributed to different operational definitions of alcohol onset^15,17^. Not quite unexpectedly, studies have also suggested that elevated risk of substance use is not substance-specific, but broader^18^. Alcohol, marijuana, and tobacco are the three most commonly used substances before age of 18^19^, and tobacco and marijuana are the two most frequently used substances in concomitant with alcohol^20,21^. The trajectory of substance use is often progressively developed in stages, from no use to initiation and escalation, ongoing to regular drinking behavior, and finally to binge drinking^22^. The reduction of binge drinking by both youths and adults is a long-lasting challenge for the United States (U.S.)^23^, and continued efforts to postpone onset of substance use are key strategies to lower the rate of adult binge drinking.

The 2013 Youth Risk Behavior Survey data indicated that among U.S. high school students who reported current use of one or more tobacco products, 70.2% reported binge drinking, 85.8% reported current alcohol drinking, and 64.5% reported current marijuana use^24^. Specifically, among youths who use tobacco, limited studies have exclusively investigated how smokeless tobacco affects binge drinking. Many people choose to use smokeless tobacco because of perceived lower risk than smoking. On the contrary, evidence has shown that approximately 3 to 4 times greater amount of nicotine is absorbed from smokeless tobacco than a cigarette, posing elevated risks among people who use smokeless tobacco^25^.

It is well-studied that binge drinking varies by demographic characteristics including age, gender, race and ethnicity^12,19,26^, socioeconomic characteristics including health insurance status, household income^23,27^, and substance use^14,16,28^. However, little is known about the patterning of early substance use in adolescence, and its implication for and the severity of binge drinking. Our study aimed to examine the main effects of the three most frequently involved substances used before age 18 and their associations with adult binge drinking (binary and severity measured in number of days in the past month), while accounting for sociodemographic and substance use-associated covariates in a general population. We expected that the early onset of different kinds of substance use was independently associated with binge drinking to various extents, and the strength of association varied by the initiation of substance use at different ages (Hypothesis 1). Importantly, early onset of substance use was hypothesized to be related with the severity of binge drinking measured by number of days of binge drinking in a month (Hypothesis 2). Further, as an indication of severe substance use, the presence of a SUD diagnosis is expected to be strongly associated with binge drinking (Hypothesis 3).

## Materials and methods

### Study design

Data were extracted from the 2018 NSDUH conducted by the Substance Abuse and Mental Health Services Administration (SAMHSA). The NSDUH is a survey that provides annual population estimates of substance use and health among the civilian non-institutionalized individuals (equal to or over 12 years old) in the U.S. Details of the survey design and data collection methods are published elsewhere^29^. The total sample size of the 2018 NSDUH data is 56,313. The current analysis was restricted to participants who aged 18 years and older which accounts for 76.4% (N = 43,026) of the full 2018 NSDUH sample. The study was exempt for review by the West Virginia University Institutional Review Board. The study was carried out in accordance with the relevant national and international guidelines. Informed consent was not applicable in this secondary data analysis.

### Measures

Dependent variables include binge drinking as a dichotomized variable as well as an ordinal variable. Participants were asked if they engaged in alcohol use and binge drinking in the past month. Binge Alcohol Frequency, IRALCBNG30D, is defined as the number of days (ranged 0 to 30 days) in the past month on which the respondent reported drinking five or more drinks on the same occasion for males or four or more drinks on the same occasion for females. For this variable, “occasion” means at the same time or within a couple hours of each other. Predictors were selected based on an association with binge drinking reported by previous literatures.

#### Demographic variables

Demographic factors included participants’ age in groups (18–25 years, 26–49 years, 50–64 years and 65 years or older), sex (male and female), and race/ethnicity (Non-Hispanic White, Non-Hispanic African Americans, Hispanics, and others). The annual income was dichotomized into lower than $49,999 and $50,000 or more. Insurance status was defined to be “Yes” if a person was cover by any of the following insurances: (1) private insurance, (2) Medicare, (3) Medicaid/CHIPCOV, (4) TRICARE, CHAMPUS, CHAMPVA, VA, or Military, and (5) other health insurance.

#### Substance use disorders (SUDs) in the past year

NSDUH defined SUDs as a combined variable of abuse or dependence on illicit drugs in the past year, if the respondent reported a positive response to three or more of the following seven dependence criteria, derived from the criteria in the American Psychiatric Association (APA) Diagnostic and Statistical Manual of Mental Disorders, 4th edition (DSM IV)^29^: 1. spent a great deal of time over a period of a month getting, using, or getting over the effects of the substance; 2. unable to keep set limits on substance use or used more often than intended; 3. needed to use substance more than before to get desired effects or noticed that using the same amount had less effect than before; 4. unable to cut down or stop using the substance every time he or she tried or wanted to; 5. continued to use substance even though it was causing problems with emotions, nerves, mental health, or physical problems; 6. reduced or gave up participation in important activities due to substance use; 7. if they had experienced substance specific withdrawal symptoms at one time that lasted for longer than a day after they cut back or stopped using. Illicit drug abuse or drug dependence is defined as abusing any of the following substances: marijuana, hallucinogens, inhalants, tranquilizers, cocaine, heroin, pain relievers, stimulants, or sedatives.

#### Serious psychological distress (SPD)

SPD is a nonspecific measure of psychological distress that has been psychometrically validated and shown to be able to discriminate community DSM-IV cases from non-cases^30^. It is intended to characterize having at least 1 mental disorder, such as major depressive disorder, generalized anxiety disorder, or schizophrenia, as well as having serious impairment of body function. SPD was determined using the Kessler 6 (K6) scale, which comprised 6 questions asking how often during the past 30 days a person felt “so sad that nothing could cheer them up,” “nervous,” “restless,” “hopeless,” “worthless,” or that “everything was an effort.” Responses were scored from 0 (none of time) to 4 (all the time) for each question and summed to produce a total score (0 to 24) of the 6 questions. A score of 13 or above was employed to define SPD^30^.

#### Multiple chronic conditions

Many chronic conditions including asthma^8^, cancer^3,4^, chronic obstructive pulmonary disease (COPD)^9^, diabetes^10^, kidney diseases^11^, heart conditions and hypertension^7^ had well-established associations with binge drinking. The aforementioned chronic conditions in the lifetime were dichotomized into “yes” or “no”. Subjects were considered to have these conditions if they responded “yes” to the questions that either a doctor or other medical professional told they had such conditions in the lifetime. Multiple chronic conditions were created by counting the number of such conditions and the indicator was coded as none, one, two or more.

#### Early onset substance use prior to age 18

This study included ages at the first use of alcohol, smokeless tobacco, and marijuana, respectively. Three variables were further categorized into four age groups: 1–12, 13–14, 15–17, and 18 or more. The NSDUH data is rich in various substance use including alcohol, tobacco, marijuana, prescription pain reliever, methamphetamine, cocaine, etc^29^. However, many of them have high correlations and cannot be modelled together due to multi-collinearity. In this study, we selected the three most frequently involved substance uses with binge drinking as the objective of early onset of substance use.

### Statistical analysis

We weighted all analyses to account for the complex survey design. All analyses were conducted using SAS 9.4 (SAS Institute, Cary, North Carolina, USA). The statistical significance level was set at 0.05.

#### Descriptive statistics and prevalence

We first calculated descriptive statistics to estimate the weighted prevalence of binge drinking, overall and by each category of covariate. The SAS PROC SURVEYFREQ was used for weighted estimation of prevalence and Chi-square test was used to compare the prevalence of binge drinking across demographic subgroups (age, gender, and race), socioeconomic factors (income level, health condition, and health insurance status), clinical factors (multiple chronic disease and SPD), and substance use associated factors (SUD, early onset of alcohol use, early onset of smokeless tobacco use, and early onset of marijuana use). We also calculated the weighted mean number of days of binge drinking in the past month across the subgroups through SAS PROC SURVEYMEANS.

#### Multivariate logistic regression analysis

The PROC SURVEYLOGISTIC was used to estimate the odds ratios (ORs) and 95% confidence intervals (CIs) for the association between factors and binge drinking. Two models were conducted in sequence following a purposeful model selection process^31^. In model I, bivariate logistic model was fitted for each aforementioned factor to assess its association with binge drinking. In model II, the weighted multivariate logistic regression was fitted to adjust for potential factors with p-value significant at 0.2 in the model one^32^. Adjusted odds ratios (aORs) and 95% CIs were reported for the selected factors in model II.

#### Poisson regression analysis

**Figure 1:**
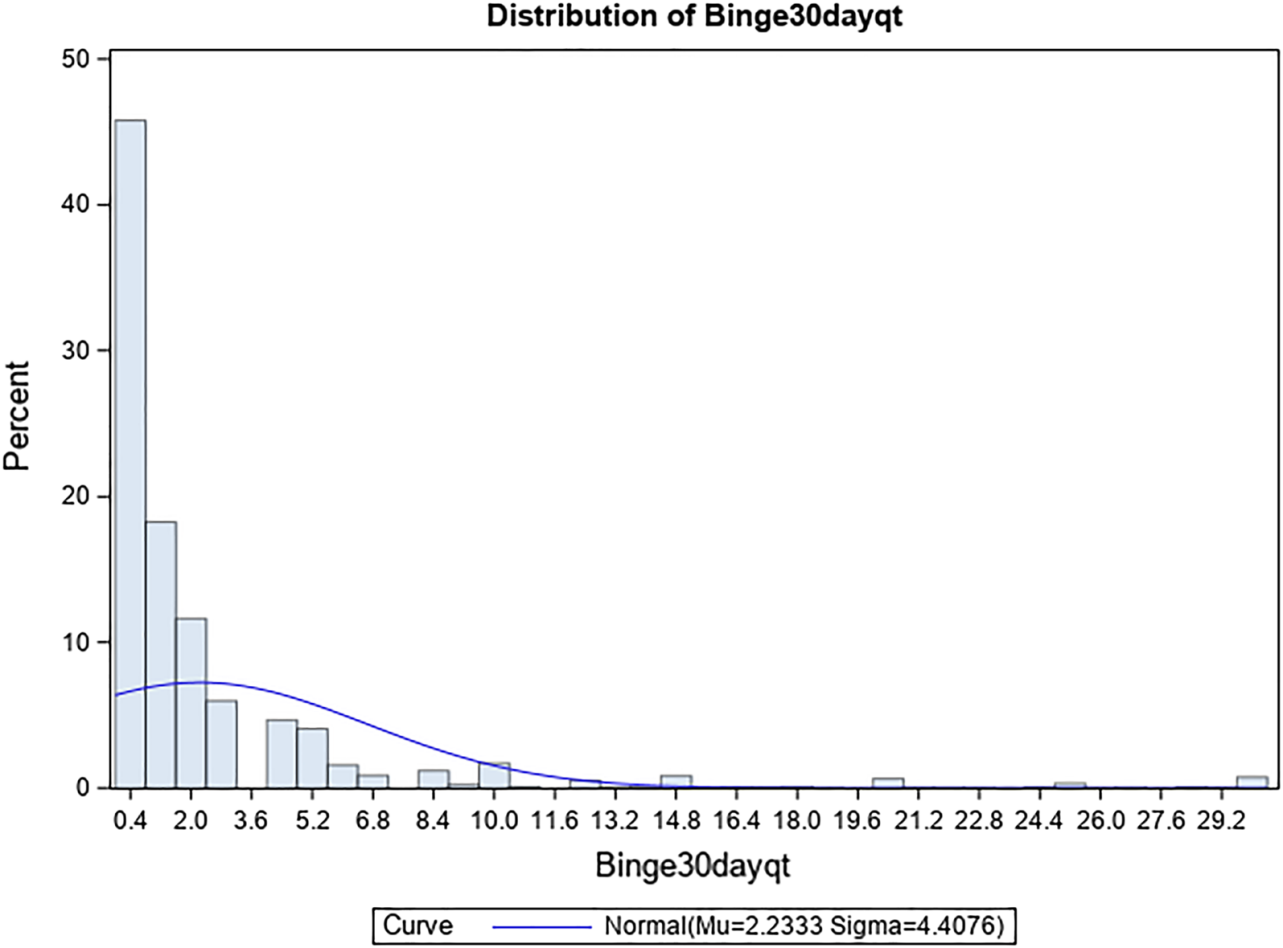
Histogram of number of days of binge drinking in the past month among 2018 NSDUH adult participants.

The PROC GLIMMIX was employed to fit a Poisson regression model to estimate the coefficients of aforementioned factors for the number of days of binge drinking in the past month^33^. This approach addressed the limitation of unable to account for sampling weight by traditional procedures of GENMOD and COUNTREG in Poisson regression. Regression coefficient (β) and corresponding standard error (SE), t-value and p-value were reported. Although zero-inflated Poisson regression seemed to be a better fit for this analysis, given that many people reported no binge drinking in the past month (Fig. 1), the SAS 9.4 cannot take both zero-inflated distribution and complicate survey weight into account at the same time. Thus, a Poisson regression model was fitted by PROC GLIMMIX.

### Institutional review board statement

The is a secondary data analysis which was exempt for review by the West Virginia University Institutional Review Board. Participants’ consents are not applicable in this study.

## Results

### Prevalence of binge drinking

The prevalence of binge drinking in the past year is listed in Table 1. Among the 43,026 adult NSDUH participants in 2018, 13,201 reported binged drinking in an average of 2.26 days in the past month. The prevalence of binge drinking is estimated to be 26.3% (95% CI 25.7–27.0%). The differences of prevalence of binge drinking were statistically significant across all factors (all p-values < 0.0001). A higher prevalence was characterized as being male (30.8 vs. 22.1% of female), aged 18–34 years (35.7% vs. ranged 30.9–10.7% in older age groups), being Hispanic (27.6%) or White (27.2%), with higher annual income than $50,000 (27.7 vs. 24.6% of lower income), in excellent or very good health condition (28.0 vs. 24.1% in poorer health), without health insurance (32.8 vs. 25.6% with health insurance), without any chronic diseases (30.5 vs. 23.1% with one and 15.5% with more than one chronic disease), with SPD (33.5 vs. 25.4% without SPD). The prevalence of binge drinking was almost two times higher in the SUD population compared to those without SUD (50.6 vs. 25.6%). All early onset of substance use subgroups had disproportionally high prevalence of binge drinking. For onset of alcohol use before age 18, the prevalence ranged from 35.8 to 44.0% with the highest occurred in the age 13–14. The highest prevalence occurred in the age 13–14 of onset of smokeless tobacco and the prevalence ranged from 39.6 to 46.7%. Among people who had first marijuana use before age 18, the prevalence ranged from 42.6 to 43.1% with the highest occurred in the age 1–12.

**Table 1:** Descriptive statistics of binge drinking across factors.

Variable	Total (N)	Binge drinking	Prevalence (%), 95% CI	p-value	Mean ± SD
**Gender**					
Male	20,169	7068	30.8 (29.9–31.7)	< 0.0001	2.50 ± 0.07
Female	22,857	6133	22.1 (21.2–23.0)		1.54 ± 0.05
**Age group (years)**					
18–34	22,431	7981	35.7 (34.8–36.7)	< 0.0001	2.34 ± 0.06
35–49	11,688	3653	30.9 (30.1–31.8)		2.18 ± 0.06
50–64	4938	1137	23.5 (22.1–24.9)		2.07 ± 0.12
65 +	3969	430	10.7 (9.3–12.0)		1.14 ± 0.11
**Race**					
White	25,834	8379	27.2 (26.3–28.0)	< 0.0001	2.14 ± 0.06
AA	5400	1438	25.4 (23.8–27.0)		1.81 ± 0.12
Hispanic	7465	2267	27.6 (26.2–29.1)		1.94 ± 0.07
Other	4237	1117	18.7 (17.0–20.4)		1.47 ± 0.08
**Income**					
< $49,999	21,257	6241	24.6 (23.6–25.6)	< 0.0001	2.45 ± 0.08
$50,000 +	21,769	6960	27.7 (26.9–28.5)		1.80 ± 0.05
**Health**					
Excellent/very good	26,011	8257	28.0 (27.1–29.0)	< 0.0001	1.78 ± 0.05
Good/Fair/poor	17,006	4942	24.1 (23.1–25.0)		2.48 ± 0.07
**Health insurance**					
No	4929	1641	32.8 (30.7–34.9)	< 0.0001	1.95 ± 0.05
Yes	38,097	11,560	25.6 (25.0–26.2)		2.92 ± 0.12
**Multiple chronic diseases**					
No	29,227	9734	30.5 (29.6–31.3)	< 0.0001	2.19 ± 0.05
One	9523	2661	23.1 (22.1–24.1)		1.89 ± 0.09
Two or more	3834	691	15.5 (13.9–17.0)		1.51 ± 0.13
**SPD**					
No	36,207	10,780	25.4 (24.7–26.1)	< 0.0001	1.95 ± 0.05
Yes	6819	2421	33.5 (31.8–35.2)		2.66 ± 0.10
**SUDs**					
No	41,206	12,243	25.6 (24.9–26.2)	< 0.0001	1.95 ± 0.04
Yes	1820	958	50.6 (47.0–54.3)		4.15 ± 0.24
**Early alcohol use (years)**					
1–12	2722	1238	39.4 (36.5–42.3)	< 0.0001	3.78 ± 0.22
13–14	4341	2090	44.0 (41.6–46.3)		3.28 ± 0.14
15–17	13,780	5732	35.8 (34.4–37.2)		2.15 ± 0.06
18 +	22,183	4141	16.3(15.7–16.9)		1.25 ± 0.05
**Early smokeless tobacco use (years)**					
1–12	806	364	39.6 (34.4–44.8)	< 0.0001	4.34 ± 0.42
13–14	956	473	46.7 (41.8–51.5)		4.93 ± 0.36
15–17	2667	1345	45.1 (42.1–48.1)		3.56 ± 0.24
18 +	38,458	10,970	24.6 (23.9–25.3)		1.80 ± 0.05
**Early marijuana use (years)**					
1–12	1414	666	43.1 (38.4–47.9)	< 0.0001	4.57 ± 0.33
13–14	3047	1418	42.7 (40.1–45.4)		3.20 ± 0.18
15–17	8375	3846	42.6 (41.0–44.2)		2.89 ± 0.10
18 +	30,190	7271	20.6 (19.9–21.4)		1.50 ± 0.05
Overall	43,026	13,201	26.3 (25.7–27.0)		2.26 ± 0.08

*AA* African American, *SPD* serious psychological distress, *SUDs* substance use disorders, *CI* confidence interval, *SD* standard deviation.

p-value is based on χ^2^ test.

The histogram in Fig. 1 depicts the frequency distribution of number of days of binge drinking in the past month. The mean and median number was 2.26 and 1 day in a month, respectively. The overall distribution is right skewed. Noteworthy, among people who reported binge drinking, on average men had one more day drinking excessively than women (2.50 vs. 1.54 days). The difference in the frequency of binge drinking at a similar level can be found between the presence and absence of health insurance (2.92 vs. 1.95 days), SPD diagnosis (2.66 vs. 1.95 days), SUD diagnosis (4.15 vs. 1.95 days), and good or poor health status (2.48 vs. 1.78 days). The highest numbers of days of binge drinking in a month were found in the subgroup of early onset of alcohol use in age 1–12 (3.78 days), 13–14 (3.28 days), early onset of smokeless tobacco use in age 1–12 (4.34 days), 13–14 (4.93 days), 15–17 (3.56 days), and early onset of marijuana use in age 1–12 (4.57 days), and 13–14 (3.20 days).

#### Weighted logistic regression analyses of binge drinking

The estimated model effects (aOR with 95% CIs and p-value) of multivariable logistic regression of independent factors are presented in Table 2. Factors that were statistically significantly associated with binge drinking as a binary variable were being male (1.33 [1.24–1.42]), being African American (1.10 [1.01–1.20]), with no multiple chronic diseases (1.49 [1.31–1.71]) or one (1.31 [1.14–1.51]), presence of SUD (1.40 [1.20–1.62]), early onset of alcohol use at age 1–12 (2.36 [2.04–2.74]), 13–14 (2.72 [2.38–3.12]), 15–17 (2.28 [2.11–2.45]), early onset of smokeless tobacco at age 13–14 (1.34 [1.09–1.65]) and 15–17 (1.24 [1.07–1.43]), and early onset of marijuana use at 1–12 (1.26 [1.01–1.58]), 13–14 (1.25 [1.09–1.44]), 15–17 (1.54 [1.42–1.66]). The largest aORs were found in the early onset of alcohol use with values larger than 2. Factors that were statistically significant protector from binge drinking include aged 35–49 (0.81 [0.76–0.87]), aged 50–64 (0.57 [0.51–0.63]), aged 65 or more (0.32 [0.28–0.38]), race being not White, Black, or Hispanic (0.68 [0.59–0.78]), with health insurance (0.90 [0.81–1.00]).

**Table 2:** Multivariable logistic regression with binge drinking as binary and Poisson regression with number of days of binge drinking in the past month as a count variable.

Variable	aOR (95%CI)	p-value	β ± SE	t, p
**Gender (ref = Female)**				
Male	1.33 (1.24–1.42)	< 0.0001	0.34 ± 0.02	22.21, < 0.0001
**Age group (ref = 18–34 years)**				
35–49	0.81 (0.76–0.87)	< 0.0001	0.003 ± 0.02	0.19, 0.8493
50–64	0.57 (0.51–0.63)	< 0.0001	− 0.02 ± 0.02	− 0.95, 0.3439
65 +	0.32 (0.28–0.38)	< 0.0001	− 0.34 ± 0.03	− 12.00, < 0.0001
**Race (ref = Whites)**				
AA	1.10 (1.01–1.20)	0.0321	− 0.18 ± 0.03	− 7.05, < 0.0001
Hispanic	1.04 (0.95–1.14)	0.3690	− 0.32 ± 0.03	− 9.74, < 0.0001
Other	0.68 (0.59–0.78)	< 0.0001	− 0.18 ± 0.02	− 8.13, < 0.0001
**Income (ref = > 50,000)**				
< 49,999	1.05 (0.98–1.13)	0.1468	− 0.28 ± 0.02	− 18.50, < 0.0001
**Health (ref = Excellent/very good)**				
Good/fair/poor	0.95 (0.88–1.03)	0.1990	0.27 ± 0.01	18.03, < 0.0001
**Health insurance (ref = No)**				
Yes	0.90 (0.81–1.00)	0.0478	− 0.11 ± 0.02	− 5.16, < 0.0001
**Multiple chronic diseases (ref = 2)**				
0	1.49 (1.31–1.71)	< 0.0001	0.30 ± 0.03	11.31, < 0.0001
1	1.31 (1.14–1.51)	0.0003	0.20 ± 0.03	7.33, < 0.0001
**SPDs (ref = No)**				
Yes	1.04 (0.96–1.13)	0.3071	0.06 ± 0.02	3.14, 0.0017
**SUDs (ref = No)**				
Yes	1.40 (1.20–1.62)	< 0.0001	0.22 ± 0.03	7.92, < 0.0001
**Alcohol use (ref = 18 +)**				
1–12	2.36 (2.04–2.74)	< 0.0001	0.60 ± 0.03	22.27, < 0.0001
13–14	2.72 (2.38–3.12)	< 0.0001	0.58 ± 0.02	24.24, < 0.0001
15–17	2.28 (2.11–2.45)	< 0.0001	0.31 ± 0.02	16.77, < 0.0001
**Smokeless tobacco use (ref = 18 +)**				
1–12	1.21 (0.95–1.53)	0.1274	0.29 ± 0.04	7.59, < 0.0001
13–14	1.34 (1.09–1.65)	0.0060	0.44 ± 0.03	13.78, < 0.0001
15–17	1.24 (1.07–1.43)	0.0041	0.24 ± 0.02	10.33, < 0.0001
**Marijuana use (ref = 18 +)**				
1–12	1.26 (1.01–1.58)	0.0434	0.47 ± 0.03	14.84, < 0.0001
13–14	1.25 (1.09–1.44)	0.0017	0.19 ± 0.03	7.55, < 0.0001
15–17	1.54 (1.42–1.66)	< 0.0001	0.31 ± 0.02	17.82, < 0.0001

*ref* reference group, *AA* African American, *aOR* adjusted odds ratio, *CI* confidence interval, *β* regression coefficient, *SE* standard error, *SUD* substance use disorder, *SPD* serious psychological distress.

### Weighted Poisson regression analyses of number of days of binge drinking

The estimated model effects (β, SE, t, p) of multivariable Poisson regression are also presented in Table 2. Almost all factors other than age were statistically associated with the frequency of binge drinking as a count outcome. The coefficient β indicates an average change in the number of days of binge drinking in the past month associated with 1 unit change of that factor. For example, the largest β of 0.60 was associated with early alcohol use before age 12, which means that on average, people who initiated alcohol use before 12 had 0.6 more day of binge drinking in a month, compared to those who had no early initiation of alcohol. Other large βs were found in the early onset of alcohol use in age 13–14 (0.58), and 15–17 (0.31), early onset of smokeless tobacco use in age 1–12 (0.29), 13–14 (0.44), and 15–17 (0.24), early onset of marijuana use in age 1–12 (0.47), 15–17 (0.31), SUD diagnosis (0.22), no multiple chronic diseases (0.30), being male (0.34), and with poorer health (0.27). The lowest βs were found in being race other than White, Black, or Hispanic (− 0.32), aged 65 or more (− 0.34), lower income (− 0.28).

## Discussion

The current study confirms how early onset of substance use affects the development of adult binge drinking behavior and expands our understanding by quantifying the impact of early onset of substance use on the severity of binge drinking. All three predictors of onset of substance use prior to age 18, including alcohol, smokeless tobacco, and marijuana, were identified as independent predictors of binge drinking and were found statistically significantly associated with the binge drinking to various extents. Early onset of alcohol use was the strongest, following smokeless tobacco and marijuana use. The current study aligns with previous findings that the earlier onset of alcohol drinking, the more likely these individuals are to develop binge drinking and other alcohol use related problems^16,34^. However, some previous studies had contradicted findings. Moss et al.^35^ found a positive association between early marijuana use and later binge drinking, but a surprisingly negative effect of early cigarettes smoking on adulthood binge drinking. The reasons for varying substance use effects are not clear but might be related to that the different distributional networks vary by substances used. Future research focused on how different patterns or sequences of early onset of substance use (e.g., initiation of alcohol use leads to tobacco use and then marijuana use) affect binge drinking may shed light on this question.

Of great importance, the current study supports that the presence of SUD diagnosis in the past year is one of the strongest predictors of binge drinking. On average people who were diagnosed with SUD last year had over two times more days of binge drinking in a month, compared to people without SUDs (4.15 vs. 1.95 days). Previous studies have shown that adolescent use of all the three substances was strongly associated with SUD diagnoses in the young adulthood^13,36–40^. The significant impact of SUD on binge drinking might be an additive or synthetic effect of the onset of experimenting with substances at an early age^35^. Thus, delaying the onset of substance use, including tobacco, alcohol, marijuana, would be an effective strategy for preventing both addiction and binge drinking in adulthood life.

Demographic characteristics including being male and in young adulthood (age 18–34) are also strongly associated with binge drinking. A national report indicated that between 2001–2002 and 2012–2013, high-risk drinking and alcohol use disorder increased across almost all sociodemographic subgroups in the U.S., especially among women, older adults, racial/ethnic minorities, and the socioeconomically disadvantaged populations^41^. Being African American is statistically significantly (p = 0.03) associated with binge drinking, which is in consistent with a previous NSDUH report^28^. The results revealed the importance of greater public health efforts to reduce binge drinking across these demographics.

Socio-economic factors are mostly marginally statistically (p-values in 0.05–0.20) associated with binge drinking, including low house income (< $49,999), relatively good health (excellent or very good), and no health insurance. These findings are not greatly contradicting with previous studies^28,42^. Alcohol consumption is well-known to be causally associated with and complicated with many chronic diseases including diabetes, heart diseases, and injuries^7–11^. However, the present study surprisingly found that higher number of chronic diseases was a protective factor of binge drinking, which aligns with a previous finding that the prevalence of binge drinking was lower among older adults with two or more chronic diseases^28^. The reason for this seemingly unjustified finding might be that patients in multiple chronic diseases are in serious health conditions and have to follow doctor’s orders to avoid alcohol consumption^28^.

### Strengths

The analysis provides a unique strength that measures the severity of binge drinking by number of days in the past month as a count outcome. To our knowledge, the study is the first to investigate risk factors associated with binge drinking as a count outcome using a weighted Poisson regression model in a nationally representative sample. Another unique strength of the current study exists in the classification of age at onset of substance use, the findings of which indicate that the influential range of age at early onset varies by substance. Onset of alcohol use at any age prior to 18 was found statistically significantly associated with the binge drinking (all ORs > 2); onset of smokeless tobacco use after 13 and onset of marijuana use at any age before 18 were also found statistically significantly related to the binge drinking (all ORs ranging from 1 to 2).

### Limitations

Despite of many strengths that have shown in this section, our study presents a series of limitations. The cross-sectional design impedes an interpretation of causational factors. The nature of NSDUH data is prone to recall bias, social desirability bias, and non-differential misclassification bias due to the collection of information on the past behaviors (e.g., past-month binge drinking and number of days of binge drinking in the past month)^20,27^. There is no worldwide consensus of definition of “binge drinking” or “heavy drinking episode”. The definition used in this study was derived from the US academia and federal agency^29,43^, and could be different from other reports or publications worldwide. Another noteworthy limitation to the interpretation of our results is that the study failed to take age of first intoxication by alcohol into consideration. While age of onset of alcohol use has been identified as an independent risk factor of binge drinking, previous studies^16,17^ suggest that delay to intoxication may be an important determinant of negative alcohol use outcome and should be considered in the modelling. Although the NSDUH includes various substance use information, the study only includes the main three substances which are the most prevalent among binge drinking population. Besides, unfortunately, this study cannot take interpersonal factors, including parental monitoring and relationships between parents and adolescents, as known risk factors into the modelling^44^.

## Conclusion

Binge drinking is prevalent among adults and its severity is significantly associated with early onset of substance use to various extents, especially with regards to early alcohol use. The findings from this study could help identify adolescents at high risk of binge drinking before age of 12, especially among those who use dual or multiple products of tobacco, alcohol, and/or marijuana. Reduced alcohol, tobacco, and marijuana use should be integrated in the brief physician advice towards adolescents with drinking problems, any substance use issues, mental health conditions and/or multiple chronic diseases. Health education that specifically emphasize delayed use of alcohol, tobacco, and/or marijuana should be conducted on campus, especially among high school students. The U.S. Preventive Services Task Force recommends alcohol screening for all adults aged at or over 18 years^14^, and it is becoming important to extend the alcohol screening among younger populations. Future research should examine how different patterns of early onset of substance use affect binge drinking while controlling for the onset of substance use.

## Data Availability

The data that support the findings of this study are publicly available at Substance Abuse & Mental Health Data Archive, the National Survey on Drug Use and Health 2018 data.

## Code availability

The codes for the statistical analysis can be requested from corresponding author via email at zheng.dai@hsc.wvu.edu. https://www.datafiles.samhsa.gov/dataset/national-survey-drug-use-and-health-2018-nsduh-2018-ds0001.
